# The Role of Sociodemographic Characteristics and Social Determinants of Health in Influencing the Perceived Quality of Patient–Provider Communication

**DOI:** 10.3390/nursrep15030113

**Published:** 2025-03-19

**Authors:** Nada Eldawy, Sahar Kaleem, Vama Jhumkhawala, Goodness Okwaraji, Samantha Jimenez, Joshua Sohmer, Maria Mejia, Panagiota Kitsantas, Lea Sacca

**Affiliations:** Charles E. Schmidt College of Medicine, Florida Atlantic University, Boca Raton, FL 33431, USA; neldawy2022@health.fau.edu (N.E.); skaleem2022@health.fau.edu (S.K.); vjhumkhawala2022@health.fau.edu (V.J.); gokwaraji2018@health.fau.edu (G.O.); jimenezs2023@health.fau.edu (S.J.); jsohmer2022@health.fau.edu (J.S.); mejiam@health.fau.edu (M.M.); pkitsanta@health.fau.edu (P.K.)

**Keywords:** patient–provider communication, social determinants of health, informed decision making, quality communication

## Abstract

**Background**: Patient-centered communication is a critical process in high-quality healthcare that emphasizes the reciprocal sharing of information between providers and patients to ensure care aligns with the patient’s needs, preferences, and personal values. A significant challenge arises from the healthcare provider’s time constraints during clinical encounters and the lack of adequate training on how to adopt a patient-centered communication style that addresses patient concerns, making it difficult to foster an environment conducive to shared decision making. These issues are further exacerbated by cultural and language barriers, along with low levels of health literacy and social determinants of health (SDoHs), which complicate efforts to deliver patient-centered care. **Objective**: This study examined quality criteria for patient–provider communication (PPC) and their associations with sociodemographic characteristics and SDoHs on housing, transportation, and food insecurity. **Methods**: This retrospective cross-sectional study analyzed data from the 2022 Health Information National Trends Survey (HINTS-6) national dataset. Associations between PPC and sociodemographic variables were tested using the chi-squared test. Binary logistic regression was carried out to examine the association between three PPC criteria and each of the sociodemographic characteristics and patient comfort in disclosing information on SDoHs. **Results**: Bivariate analyses showed statistically significant associations for age, occupation status, marital status, Hispanic origin, and race across all three PPC criteria. Significant associations were reported for education and income for the two criteria related to being given the chance to ask questions and being involved in healthcare decisions. Finally, significant associations were reported for all PPC criteria and patient comfort levels in discussing SDoHs. **Conclusions**: Findings from this paper provide insight for enhancing the quality of PCC in underserved populations, particularly when it comes to informing the design of evidence-based cervical cancer screening interventions which are culturally centered around the patients’ needs and that integrate PPC as a foundational component.

## 1. Introduction

Patient-centered communication is a critical process in high-quality healthcare that emphasizes the reciprocal sharing of information between providers and patients to ensure care aligns with the patient’s needs, preferences, and personal values [1,2]. The foundation of this process rests on a comprehensive education provided by the clinician and an active, two-way discussion about available treatment options and potential outcomes to empower patients to make the choice that best suits their individual circumstances [1]. Building trust and respect allows the patient to feel comfortable in expressing themselves and asking questions freely when communicating with their providers about their health concerns [3]. A key element of effective decision making is the establishment of trust and mutual respect, which encourages patients to openly express their concerns and ask questions without hesitation. Because of this, patient–provider communication (PPC) should be intentional, allowing sufficient time for thorough discussion without patients feeling rushed or dismissed by their provider. When patients feel understood and genuinely heard, they are more likely to engage actively in the decision-making process [3].

Several communication strategies can be employed to foster this level of engagement. Research suggests that providers should incorporate the use of affiliation words (e.g., “together” and “help”) to build rapport and a sense of collaboration with the patient. Additionally, using causation words (e.g., “because” and “based on”) and differentiation words (e.g., “but” and “except”) can facilitate patients’ understanding of complex medical information. Conversely, minimizing the use of insight words (e.g., “know” and “realize”) can help avoid an authoritarian tone, thus promoting a more egalitarian dynamic in the conversation and helping to maintain professionalism while conveying respect and encouraging patient participation [4]. The benefits of patient-centered communication extend beyond establishing an interpersonal relationship with their providers, and they have been shown to directly impact health outcomes, particularly in the realm of preventative care and screening [5]. For example, in breast cancer screening, integrating the use of tools like the Breast Cancer Risk Estimator-Decision Aid (BCaRE-DA) with active PPC techniques helps to better equip patients to make informed decisions regarding mammography and the risks and benefits of screening, which can ultimately lead to early detection and improved prognosis in breast cancer [6]. Also, when patients understand the benefits and risks of screening through clear communication, they are more likely to utilize preventive services [7].

Patient-centered communication is central to patient-centered care, yet several barriers impede its effective implementation. A significant challenge arises from healthcare provider’s time constraints during clinical encounters and the lack of adequate training on how to adopt a patient-centered communication style that addresses patient concerns, making it difficult to foster an environment conducive to shared decision making [8,9]. These issues are further exacerbated by cultural and language barriers, along with low levels of health literacy, which complicate efforts to deliver patient-centered care [8]. Patients from underserved communities with pre-existing challenges in navigating the healthcare system face heightened communication barriers, which can interfere with information processing, emotional response, and taking behavioral action [10]. For patients receiving a diagnosis of life-threatening conditions, such as cancer, communication barriers may be compounded by cultural and emotional factors. For example, culturally specific emotional responses to distressing news may prevent patients from effectively communicating their concerns or asking questions about their new diagnosis and treatment options [8]. During such critical moments, patients also struggle to retain important information regarding next steps in their care [11]. Furthermore, a patient’s lack of assertiveness or limited experience with the healthcare system may find it difficult to actively enhance the patient-centered care process, contributing to dissatisfaction with their care and overall poorer health outcomes [8]. Some minority groups, particularly those with limited English language proficiency or health literacy, may initially prefer a more provider-driven communication process because they have a harder time interrupting the conversation or fully digesting medical information during the encounter [12].

In addition to language and cultural factors, social determinants of health (SDoHs) further compound barriers to PPC. SDoHs encompass everyday non-medical factors rooted in individual experiences and environments that shape health outcomes [13]. The U.S. Department of Health and Human Services Healthy People 2030 project categorizes SDoHs into economic stability, education access and quality, healthcare access and quality, neighborhood and built environment, and social and community context [14]. For example, individuals living in food deserts with poor nutritional access can experience higher rates of morbidity, a lack of education or literacy skills may create increased challenges in understanding health-related information, inadequate healthcare access can lead to delayed intervention, and a lack of social support can cause elevated stress, further contributing to poor health outcomes [15,16,17].

Language barriers are particularly pronounced among individuals with limited English proficiency or who speak English as a second language, and these patients often exhibit increased social needs directly related to SDoHs [18,19]. These patients may experience differential access to care, decreased utilization of the healthcare system, and poorer health outcomes when compared to their English-proficient counterparts [20]. Even when adjustments are made to accommodate patients’ preferred modes of communication, perceptions of care can shift, which may ultimately influence health decisions and outcomes [19]. Addressing these disparities is critical for providing equitable and accessible care, as effective communication and addressing language barriers lies at the core of improving patient–provider interactions [21].

Using data from the Health Information National Trends Survey (HINTS), this study aimed to explore associations between quality criteria for patient-centered communication and sociodemographic variables. It also examines the association between patient comfort in disclosing information on affording and accessing food, transportation issues, and housing issues and the three patient–provider communication criteria (spending enough time with provider, having the opportunity to ask all health-related questions, and being involved in healthcare decisions). By examining the interplay between communication quality, accessibility, and equity, this research seeks to provide actionable insights for enhancing PCC in underserved populations. Ultimately, the findings aim to inform strategies that improve healthcare interactions, promote informed decision making, and reduce disparities in care delivery.

## 2. Methods

Data for this study were obtained from the 2022 HINTS 6 survey, which is a nationally representative survey conducted by the National Cancer Institute [22]. The survey targets civilian, non-institutionalized adults aged 18 and above living in the United States, collecting information on health-related needs, access, behaviors, perceptions, and knowledge. The 2022 HINTS survey included new items to measure SDoHs related to housing, food insecurity, transportation, and healthcare accessibility, affordability, and quality. These items aimed to understand the impact of SDoHs on national health outcomes.

### 2.1. Survey Administration and Sample Selection

The HINTS 6 survey, administered between 7 March and 8 November 2022, utilized a mixed-mode data collection method, allowing respondents to complete the survey either online or on paper. To address low response rates, a fourth mailing with increased incentives was conducted. The survey used a two-stage sampling design: first selecting a stratified sample of residential addresses and then selecting one adult from each household. Geographic stratification was employed to increase the number of rural cases. The overall weighted response rate was 28.1% with a total sample size of 6252 respondents [22]. Missing data for the demographic variables considered in this study ranged from 1.6% to 11.7%.

### 2.2. Measures

Questions were selected from the HINTS 6 survey related to patient perception of the quality of patient–provider communication. For the purpose of our study, the quality of patient–provider communication was assessed based on time spent with doctors, involvement in healthcare decisions, and the ability to have all questions answered and concerns addressed by the provider. This was measured using three items related to communication with doctors, nurses, or other health professionals in the past 12 months: (1) How often did they spend enough time with you?; (2) How often did they involve you in decisions about your health care as much as you wanted?; and (3) How often did they give you the chance to ask all the health-related questions you had? These questions were answered on a 4-point Likert scale ranging from “Always” to “Never”. Questions were also selected relating to the role of SDoHs on PPC, which was measured by responses to whether participants would be comfortable sharing their information with their healthcare providers regarding the following: (1) issues with affording or accessing health food; (2) issues with transportation that make traveling to work or medical appointments difficult; and (3) issues with housing (for example, concerns about eviction, making mortgage payments, lead paint, or asbestos). Respondents were asked to rate their comfort level on a 4-point Likert scale ranging from “Very Comfortable” to “Very Uncomfortable”.

The sociodemographic variables used for this study included age (18–39, 40–64, >65); gender (male, female); occupation status (employed, homemaker, student, retired, disabled, unemployed, multiple occupation statuses or other occupation); marital status (married or living as married with a romantic partner, divorced or separated, widowed, single never been married); educational level (<8 years, 8–12 years, post high school training or some college, college graduate, postgraduate); Hispanic origin (not Hispanic or Hispanic); race (White, Black, American Indian or Alaska Native, Asian, other Pacific Islander); and income level ($0 to $49,999; $50,000–$99,999; $100,000–$199,999; $200,000 or more).

Several sociodemographic variables were adjusted by combining sub-categories to avoid small cell sizes for the purpose of analysis. Age was originally a continuous variable but was recoded into a categorical variable with three sub-categories (18–39 years; 40–64 years; and ≥65 years). For Occupation Status, “Homemaker”, “Student, “Retired”, and “Disabled” were combined in one category, and “Unemployed for less than year” was combined with “Unemployed for >1 year” into a single “Unemployed” category. Marital status was altered by combining “Married” with “Living as married with a romantic partner”, and “Divorced” with “Widowed” and “Separated”. Combined education categories included: “8–12 years” and “post-high school” under “High School/Post-High School”, and “College graduate” and post-graduate” into one category “College Graduate/Post-Graduate”. Hispanic Origin was altered into a binomial response (Yes/No) by combining responses listed as Mexican, Puerto Rican, Cuban, other Hispanic, and Multiple Hispanic ethnicities under the “Yes” category. Asian ethnicities (Asian Indian, Chinese, Filipino, Vietnamese, other Asian) were combined into a single racial category (Asian). For income level, the original brackets were simplified into four ranges: $0 to $49,999; $50,000 to $74,999; $75,000–$99,999; $100,000 to $199,999; and $200,000 or more.

Additionally, the 4-point Likert scale, used for the three PPC quality criteria items, ranging from “Always” to “Never” was recoded into two categories (combining “Always/Usually” into one category and “Sometimes/Never” into a separate category) for each of the three items on PPC: (1)“Spent Sufficient Time”/“Spent Insufficient Time with Doctor”; (2) “Involved in Healthcare Decision”/“Not Involved in Healthcare Decision”; and (3) “Given Chance to Ask Questions”/“Not Given the Chance to Ask Questions”. This also controlled for small cell sizes for the purpose of descriptive and regression analysis.

### 2.3. Statistical Analyses

Descriptive statistics were first conducted to explore sociodemographic characteristics and patient–provider communication measures in this sample. Associations between patient-centered communication and sociodemographic variables were tested using the chi-squared test. Next, binary logistic regression was carried out to examine the association between three patient-centered communication criteria and sociodemographic characteristics and the association between patient comfort in disclosing information on affording and accessing food, transportation issues, and housing issues and the three patient–provider communication criteria. Data analysis was carried out using STATA/SE 17.0. Replicate weights were used to adjust for the complex population-based survey design. All values listed as “Missing data (not ascertained)”, “Missing data (web partial—question never seen)”, “Multiple responses selected in error”, or “Question answered in error (commission error)” were recoded to System Missing and were dropped from consideration for the purpose of analysis.

## 3. Results

### 3.1. Participant Characteristics

A total of 6,252 participants participated in the survey. However, after dropping participants with missing or incomplete information, a total of 4,348 participants were retained for analysis. More than half of the respondents were female (61.22%), White (71.92%), and non-Hispanic (85.63%). The majority of respondents had some college or a post-graduate degree (71.36%). Approximately half of the respondents had a combined household income level above $50,000 (59.63%) with 30.45% reporting an income between $50,000 and $99,999, 20.12% between $100,000 and $199,999, and 9.06% reporting $200,000 or more. Table 1 includes all characteristics for the study sample.

### 3.2. Binary Logistic Regression of the Association Between Sociodemographic Variables and the Three Criteria on Quality of Patient–Provider Communication

Three survey items were related to quality of patient–provider communication by looking at time spent with provider, ability to ask questions, and being involved in healthcare decisions. These were answered on the recoded binary scales of “Spent Sufficient Time”/“Spent Insufficient Time with Doctor”; “Involved in Healthcare Decision”/“Not Involved in Healthcare Decision”; and “Given Chance to Ask Questions”/“Not Given the Chance to Ask Questions”. Using a chi-squared test, bivariate analyses showed statistically significant associations (*p*-value < 0.05) for age, occupation status, marital status, Hispanic origin, and race across all three PPC criteria (Table 2, Table 3 and Table 4). Additionally, significant associations were reported for education and income for the two criteria on given the chance to ask questions (Table 3) and being involved in healthcare decisions (Table 4). A series of binary logistic regressions were also carried out to identify sociodemographic predictors of quality patient–provider communication based on the three assessed criteria. Results showed that participants in the 40–64 years old and >65 years old categories had (OR = 0.629; 95% CI (0.529–0.748)) and (OR = 0.453; 95% CI (0.361–0.525)) lower odds of spending insufficient time with their healthcare provider, respectively, compared to the 18–39 years old reference group category (Table 2). Similarly, both age groups had lower odds of not having the chance to ask questions (*p*-value = 0.000) (Table 3) and not being involved in healthcare decisions (*p*-value = 0.005) (Table 4) compared to their younger counterparts in the reference group category. When it comes to occupation status, those classified in the homemaker, student, retired, or disabled category had lower odds (OR = 0.626; 95% CI (0.532–0.736)) of spending insufficient time with their doctors (Table 2) and lower odds (OR = 0.677; 95% CI (0.534–0.856) of not having the chance to ask questions (Table 3) compared to their employed counterparts. On the other hand, unemployed respondents (OR = 1.792; 95% CI (1.241–2.587)) and those with multiple occupations (OR = 1.481; 95% CI (1.106–1.983)) had higher odds of not having the chance to ask questions. Additionally, those with multiple occupations (OR = 1.894; 95% CI (1.473–2.437)) had higher reported odds of not being involved in healthcare decisions (Table 4).

Moreover, divorced, widowed, or separated participants had (OR = 0.782; 95% CI (0.661–0.926)) lower odds of spending insufficient time with their doctor and higher odds of not being involved in healthcare decisions (OR = 1.258; 95% CI (1.035–1.530)), while single participants had higher odds of spending insufficient time with their doctor (OR = 1.236; 95% CI (1.032–1.480)) (Table 2), not having the opportunity to ask all health-related questions (OR = 1.457; 95% CI (1.141–1.860)) (Table 3), and not being involved in health-related decisions (OR = 1.350; 95% CI (1.082–1.686)) (Table 4) compared to their married counterparts. When it comes to education, respondents with a high school degree or higher had lower odds of not having the opportunity to ask all health-related questions (Table 3) and not being involved in healthcare decisions (Table 4) (*p*-value = 0.000). Furthermore, compared to non-Hispanic respondents, Hispanic participants had higher odds of spending insufficient time with their doctors (OR = 1.674; 95% CI (1.393–2.012)) (Table 2) along with higher odds of not being able to ask all health-related questions (OR = 1.912; 95% CI (1.513–2.434)) and not being involved in healthcare decisions (OR = 1.984; 95% CI (1.609–2.447)) (Table 4).

Interestingly, respondents identifying as Asians, American Indian/Alaska Natives, or as having multiple races were all reported to have higher odds of spending insufficient time with their doctors (Table 2), not having the chance to ask all health-related questions (Table 3), and not being involved in healthcare decisions (Table 4) compared to their White counterparts (*p*-value = 0.000). Finally, for income, respondents classified in categories earning equal to or more than $75,000 had lower odds of reporting not having the chance to ask all health-related questions (Table 3) and not being involved in healthcare decisions (Table 4) compared to the reference category ($0–$49,000). Gender, education, and income were not reported to have significant associations with the criteria of sufficient time spent with providers.

### 3.3. Binary Logistic Regression of the Association Between Patient Comfort in Discussing Food Accessibility, Transportation Difficulties, and Housing Concerns, with Three Patient–Provider Communication Criteria

A series of binary logistic regression was conducted to explore the association between comfort discussing various SDoHs (food affordability and accessibility, housing concerns, and transportation difficulties) and the three PPC criteria (Table 5). Significant associations were reported for all criteria across all levels of the 4-point Likert scale (Very Comfortable–Very Uncomfortable) used to measure patient comfort levels in discussing SDoHs. Higher odds of spending insufficient time with the doctor, not having the chance to ask questions, and not being involved in health-related decisions were reported for participants feeling very uncomfortable, somewhat uncomfortable and somewhat comfortable when it comes to disclosing issues related to food accessibility compared to those who felt very comfortable talking about such issues (*p*-value < 0.001). Similarly, higher odds of spending insufficient time with the doctor, not having the chance to ask questions, and not being involved in health-related decisions were reported for participants feeling very uncomfortable, somewhat uncomfortable and somewhat comfortable when it comes to disclosing issues related to transportation difficulties (*p*-value < 0.001) and housing concerns (*p*-value < 0.001).

## 4. Discussion

The study utilized data from the national survey HINTS-6 to examine barriers to perceived patient-centered communication and its associations with social determinants of health and patient comfort discussing non-medical factors such as food security, housing, and transportation. PCC in this study was operationalized using three key criteria: time spent with providers, involvement in healthcare decisions, and the opportunity to ask health-related questions. The findings highlight significant disparities in the quality of PCC with implications for improving health equity and patient engagement.

Certain demographic groups consistently reported lower-quality PCC across multiple criteria, including younger adults (18–39 years), individuals without stable income (e.g., unemployed), and those with lower educational attainment (less than a high school degree). The findings align with the existing literature while offering nuanced insights into the barriers faced by underserved populations [23]. Younger adults were particularly less likely to report being adequately involved in healthcare decisions. This finding is consistent with prior studies showing that younger patients (18–34 years) have the lowest-rated PCC experiences compared to older adults [23], which might reflect differences in expectations or providers’ assumptions about younger patients’ healthcare needs. Such disparities highlight the need for interventions that focus on engaging younger patients and addressing implicit gender biases in provider communication.

Household income emerged as a significant factor for two PCC quality criteria in this study, which aligns with prior literature highlighting the role of socioeconomic factors such as income, health literacy, and education in influencing PPC. Patients with lower literacy skills are less likely to ask questions or seek additional services, which can impact their overall satisfaction with provider communication [24,25]. Provider-related barriers, such as implicit biases, time constraints, and inadequate cultural competency training, further contribute to disparities in PCC [26]. Implicit biases may influence providers’ perceptions and interactions with patients, particularly those from minority or underserved populations [27,28]. Time constraints and complex cases can also limit opportunities for meaningful dialogue, impeding the development of trust and rapport [11]. Addressing these challenges requires systemic changes, such as incentivizing providers to undergo cultural competency training, extending visit durations for patients with complex needs, and incorporating SDoHs into clinical workflows [29,30,31,32,33,34].

Differences in linguistic styles and communication norms associated with socioeconomic backgrounds may exacerbate the discordance between patients and providers, further diminishing PCC quality [34,35]. Patient comfort discussing SDoHs-related issues, such as food security, housing, and transportation, was associated with PCC quality. Participants who felt uncomfortable discussing these concerns were less likely to report positive PCC experiences. Specifically, discomfort in addressing housing issues, transportation issues, and ability to access and afford food were all significantly associated with lower odds of perceiving sufficient time with providers, opportunities to ask questions, and involvement in healthcare decisions. These findings suggest that reluctance to share sensitive information may limit open communication, hindering providers’ ability to deliver holistic, patient-centered care. Normalizing discussions around SDoHs through routine screening and connecting patients to community resources could improve trust and enhance communication quality [35,36,37,38,39,40,41,42,43]. Educational attainment also plays a critical role with higher education levels associated with more comprehensive discussions and greater patient satisfaction [43]. While insurance status was not assessed in this study, prior research suggests it likely influences PCC indirectly through access to care and trust in providers [23,33]. Interventions aimed at simplifying communication and empowering patients with low literacy or education levels are essential for addressing these gaps.

Ethnic disparities were also pronounced, particularly among Hispanic, Asian, and American Indian/Alaska Native participants. Asian respondents reported significantly lower PCC quality across all criteria, which is consistent with prior research documenting the compounded effects of language barriers, cultural differences, and systemic biases in healthcare settings [37,38,39,40,41]. A 2024 study found that perceptions of everyday racism significantly influenced the quality of communication among Asian Americans, highlighting the complex interplay of cultural identity and healthcare experiences [41]. Hispanic participants were also less likely to feel adequately involved in healthcare decisions, aligning with evidence that language discordance and limited English proficiency contribute to poorer PCC experiences among Spanish-speaking Hispanic patients [43,44,45]. Regardless of the contribution of English proficiency, there appears to be a consensus that Hispanic patients receive worse quality patient-centered communication compared to non-Hispanic White patients [39,44,45]. Moreover, American Indian/Alaska Native patients were more likely to report distrust of providers due to there being a lack of effective communication that integrates their cultural values, long waiting times, and low visit expectations that do not satisfy the patient’s needs [41]. These findings underscore the importance of culturally tailored interventions, such as professional interpreter services and provider training on cultural competence, to improve communication with minority groups.

Effective strategies and interventions designed to integrate the foundational components of PPC while taking into consideration the patients’ SDoHs and health literacy levels have been implemented and consistently refined to address a diverse range of health outcomes [41,44,45,46]. For instance, in a cross-sectional survey administered to a sample of diverse, low-income patient populations attending a healthcare center for the management of cholesterol levels, different components of patient–provider communication were reported to influence patient adherence to lifestyle modification advice and medication prescription [44]. Specifically, adherence to medication was associated with having a provider who is knowledgeable about one’s medical history, while criteria such as knowledge of medical history, spending enough time, and providing easily understandable information were significantly associated with reports of following physical activity advice [44]. Knowledge of medical history and providing easily understandable information were also significantly associated with reports of following weight management advice [44]. Moreover, interventions tailored to vulnerable population groups, such as hospitalized children, were also seen to significantly impact patient satisfaction with care received [45]. One example is the Passport Program, which is a patient–provider communication program for hospitalized minority patients aiming to improve healthcare delivery and minimize disparities in care for minorities [45]. Most of the common themes reflecting patient satisfaction with care received included the organization of medical care, emotional expressions about the hospitalization experience, and overall understanding of the process of care [45]. Similar satisfaction levels were reported by both Spanish and English-speaking families; however, the Passport Program families reported improved quality of communication with the medical care team [45]. Furthermore, a systematic review exploring the impact of 73 interpersonal interventions to attain the quadruple aim of healthcare highlighted that patient-centered interactions improved provider well-being, burnout, stress, and confidence in communicating with difficult patients [41,46,47]. When it comes to cancer screening, a national survey on U.S. adults highlighted significant associations between having adult patients involved in decision making by their healthcare providers and higher odds of breast, cervical, and colorectal cancer screenings [48]. Hence, tailored approaches to optimize the impact of patient–provider communication are needed in clinical settings for decreased disparities in improved health outcomes.

### Limitations

A major strength of this study lies in its use of a nationally representative dataset, providing insights into PCC across diverse populations. However, the cross-sectional design limits causal inferences, and the reliance on self-reported data may introduce social desirability bias. Additionally, the sample’s demographic composition, predominantly non-Hispanic White, may underrepresent the experiences of minority populations and my lead to selection bias. Moreover, the HINTS survey categorizes participants as “Hispanic” and “Non-Hispanic” without justifying this classification. Furthermore, it does not explicitly analyze language proficiency as a factor, which could have provided deeper insight into communication barriers among patients with limited English proficiency. Finally, the survey does not have specific items measuring patient health status in relation to patient–provider communication criteria. Future research should focus on more diverse samples to better capture the full spectrum of PCC experiences, particularly among populations with significant access barriers. More efforts are needed to expand on survey items assessed in HINTS, particularly when it comes to specific measures related to language barriers and PPC quality.

## 5. Conclusions

This study underscores the importance of understanding the sociodemographic and social determinants influencing PCC to improve health equity. Addressing disparities in PCC requires targeted interventions that enhance communication quality for underserved groups, normalize discussions around SDoHs, and equip providers with the tools to navigate cultural and linguistic differences. Further research should explore the intersectionality of factors such as race, gender, and income to identify tailored strategies for improving patient–provider interactions and fostering more inclusive healthcare environments. The findings from this study have a significant implication for clinical practice by informing the design and adaptation of effective strategies to improve the overall quality of PPC and enhance the adoption of and adherence to cervical cancer prevention measures.

## Figures and Tables

**Table 1:** Participant demographic characteristics.

Demographic Variables	Weighted Frequency (Weighted %)
**Age**	
18–39	937 (21.55%)
40–64	1870 (43.01%)
>65	1541 (35.44%)
**Gender**	
Male	1686 (38.78%)
Female	2662 (61.22%)
**Occupation Status**	
Employed	2146 (49.36%)
Homemaker/Student/Retired/Disabled	1455 (33.46%)
Unemployed	232 (5.34%)
Multiple Occupation Statuses	450 (10.35%)
Other Occupation	65 (1.49%)
**Marital Status**	
Married/Living as Married or Living with Partner	2312 (53.17%)
Divorced/Widowed/Separated	1230 (28.29%)
Single	806 (18.54%)
**Education**	
Less than High School	211 (4.85%)
High School/Post-High School	1034 (23.78%)
Some College	921 (21.18%)
College Graduate/Post-Graduate	2182 (50.18%)
**Hispanic**	
Yes	625 (14.37%)
No	3723 (85.63%)
**Race**	
White	3127 (71.92%)
Black	749 (17.23%)
American Indian or Alaska Native	46 (1.06%)
Multiple Races	166 (3.82%)
Asian	221 (5.08%)
Pacific Islander	39 (0.90%)
**Income level**	
0–$49,999	1755 (40.36%)
$50,000–$74,999	756 (17.39%)
$75,000–$99,999	568 (13.06%)
$100,000–$199,999	875 (20.12%)
Greater or equal to $200,000	394 (9.06%)

**Table 2:** Binary logistic regression of the association between sociodemographic variables and sufficient time spent with providers.

Demographic Characteristic	Categories	Spent Sufficient Time with Doctor	Spent Insufficient Time with Doctor	*p*-Value *	OR (95% CI) Spent Insufficient Time with Doctor **
Age	18–39	630	307	0.00 *	Ref ***
	40–64	1435	435		0.629 * (0.529–0.748)
	>65	1271	270		0.435 * (0.361- 0.525)
Gender	Male	1286	400	0.577	Ref
	Female	2050	612		0.935 (0.812–1.078)
Occupation Status	Employed	1597	549	0.000 *	Ref
	Homemaker/ Student/Retired/Disabled	1201	254		0.626 * (0.532–0.736)
	Unemployed	175	57		0.946 (0.691–1.294)
	Multiple Occupation Statuses	320	130		1.180 (0.944–1.475)
	Other Occupation	43	22		1.436 (0.855–2.412)
Marital Status	Married/Living as Married or Living with Partner	1762	550	0.000 *	Ref
	Divorced/Widowed Separated	993	237		0.782 * (0.661–0.926)
	Single	581	225		1.236 * (1.032–1.480)
Education	Less than High School	149	62	0.068	Ref
	High School/Post-High School	806	228		0.671 * (0.484–0.931)
	Some College	722	199		0.674 * (0.484–0.939)
	College Graduate/Post-Graduate	1659	523		0.761 (0.559–1.037)
Hispanic	No	2911	812	0.000 *	Ref
	Yes	425	200		1.674 * (1.393–2.012)
Race	White	2437	690	0.001 *	Ref
	Black	582	167		1.046 (0.866–1.262)
	American Indian or Alaska Native	29	17		2.026 * (1.111–3.695)
	Multiple Races	115	51		1.558 * (1.11–2.188)
	Asian	145	76		1.860 * (1.395–2.480)
	Pacific Islander	28	11		1.405 (0.696–2.835)
Income level	0–$49,999	1388	417	0.076	Ref
	$50,000–$74,999	574	182		1.031(0.846–1.256)
	$75,000–$99,999	419	149		1.123 (0.906–1.391)
	$100,000–$199,999	699	176		0.816 * (0.671–0.993)
	Greater or equal to $200,000	306	88		0.929 (0.718–1.203)

* The *p*-value (based on chi-squared test) tests for associations between sociodemographic variables and sufficient time spent with providers. ** Category model is predicting. *** Reference category.

**Table 3:** Binary logistic regression of the association between sociodemographic variables and opportunity to ask all health-related questions.

Demographic Characteristic	Categories	Had the Chance to Ask Questions	Did Not Have the Chance to Ask Questions	*p*-Value *	OR (95% CI) Did Not Have The Chance to Ask Questions **
Age	18–39	811	126	0.000 *	Ref ***
	40–64	1668	202		0.789 * (0.623–0.999)
	>65	1426	115		0.502 * (0.385–0.654)
Gender	Male	1515	171	0.936	Ref
	Female	2390	272		1.008 (0.821–1.223)
Occupation Status	Employed	1923	223	0.000 *	Ref
	Homemaker/ Student/Retired/Disabled	1348	107		0.677 * (0.534–0.856)
	Unemployed	192	40		1.792 * (1.241–2.587)
	Multiple Occupation Statuses	385	65		1.481 * (1.106–1.983)
	Other Occupation	57	8		1.178 (0.556–2.497)
Marital Status	Married/Living as Married or Living with Partner	2092	220	0.008 *	Ref
	Divorced/Widowed Separated	1114	116		0.999 (0.791–1.262)
	Single	699	107		1.457 * (1.141–1.860)
Education	Less than High School	168	43	0.000 *	Ref
	High School/Post-High School	921	113		0.480 * (0.327–0.703)
	Some College	821	100		0.468 * (0.317–0.691)
	College Graduate/Post-Graduate	1995	187		0.365 * (0.254–0.525)
Hispanic	No	3382	341	0.000 *	Ref
	Yes	523	102		1.912 * (1.513–2.434)
Race	White	2846	281	0.001 *	Ref
	Black	663	86		1.359 * (1.057–1.747)
	American Indian or Alaska Native	35	11		3.142 * (1.582–6.240)
	Multiple Races	144	22		1.539 (0.967–2.449)
	Asian	184	37		2.003 * (1.380–2.906)
	Pacific Islander	33	6		1.870 (0.777–4.500)
Income level	0–$49,999	1525	230	0.016 *	Ref
	$50,000–$74,999	669	87		0.873 (0.673–1.132)
	$75,000–$99,999	519	49		0.610 * (0.442–0.843)
	$100,000–$199,999	826	49		0.402 * (0.293–0.550)
	Greater or equal to $200,000	366	28		0.500 * (0.332–0.751)

* The *p*-value (based on chi-squared test) tests for associations between sociodemographic variables and opportunity to ask all health-related questions. ** Category model is predicting. *** Reference category.

**Table 4:** Binary logistic regression of the association between sociodemographic variables and patient involvement in healthcare decisions.

Demographic Characteristic	Categories	Involved in Decision	Not Involved in Decision	*p*-Value *	OR (95% CI) Not Involved in Decision **
Age	18–39	777	160	0.004 *	Ref ***
	40–64	1610	260		0.792 * (0.640–0.981)
	>65	1351	190		0.680 * (0.543–0.852)
Gender	Male	1466	220	0.136	Ref
	Female	2272	390		1.104 (0.927–1.315)
Occupation Status	Employed	1865	281	0.000 *	Ref
	Homemaker/ Student/Retired/Disabled	1280	175		0.910 (0.746–1.109)
	Unemployed	194	38		1.287 (0.890–1.862)
	Multiple Occupation Statuses	350	100		1.894 * (1.473–2.437)
	Other Occupation	49	16		2.083 * (1.172–3.703)
Marital Status	Married/Living as Married or Living with Partner	2019	293	0.016 *	Ref
	Divorced/Widowed Separated	1045	185		1.258 * (1.035–1.530)
	Single	674	132		1.350 * (1.082–1.686)
Education	Less than High School	161	50	0.001 *	Ref
	High School/Post-High School	880	154		0.571 * (0.400–0.815)
	Some College	781	140		0.579 * (0.404–0.831)
	College Graduate/Post-Graduate	1916	266		0.446 * (0.318–0.625)
Hispanic	No	3253	470	0.000 *	Ref
	Yes	485	140		1.984 * (1.609–2.447)
Race	White	2728	399	0.001 *	Ref
	Black	638	111		1.218 (0.974–1.522)
	American Indian or Alaska Native	32	14		2.909 * (1.543–5.481)
	Multiple Races	135	31		1.551 * (1.036–2.322)
	Asian	173	48		1.859 * (1.330–2.600)
	Pacific Islander	32	7		1.500 (0.658–3.42)
Income level	0–$49,999	1441	314	0.000 *	Ref
	$50,000–$74,999	637	119		0.864 (0.687–1.085)
	$75,000–$99,999	505	63		0.562 * (0.422–0.748)
	$100,000–$199,999	800	75		0.430 * (0.331–0.560)
	Greater or equal to $200,000	355	39		0.505 * (0.357–0.715)

* The *p*-value (based on chi-squared test) tests for associations between sociodemographic variables and patient involvement in healthcare decisions. ** Category model is predicting. *** Reference category.

**Table 5:** Binary logistic regression of the association between patient comfort in discussing food accessibility, housing concerns, and transportation difficulties with three patient–provider communication criteria.

Patient Comfort Sharing Information	Time Spent with Doctor aOR ** (95% CI)		Involvement in Health-Related Decisions aOR (95% CI)		Chance to Ask Questions aOR (95% CI)	
**Affording and Accessing Healthy Food**						
Very Comfortable	Ref ***	***p*-value**	**Ref**	***p*-value**	**Ref**	***p*-value**
Somewhat Comfortable	1.354 (1.120–1.636)	<0.001 *	1.408 (1.112–1.782)	<0.001 *	1.546 (1.165–2.052)	<0.001 *
Somewhat Uncomfortable	1.808 (1.480–2.207)		1.802 (1.409–2.305)		2.180 (1.633–2.909)	
Very Uncomfortable	1.775 (1.443–2.184)		1.849 (1.435–2.383)		2.316 (1.725–3.109)	
**Transportation Issues**						
Very Comfortable	**Ref**	***p*-value**	**Ref**	***p*-value**	**Ref**	***p*-value**
Somewhat Comfortable	1.299 (1.083–1.557)	<0.001 *	1.296 (1.033–1.625)	<0.001 *	1.522 (1.167–1.984)	<0.001 *
Somewhat Uncomfortable	1.725 (1.416–2.101)		1.772 (1.393–2.255)		2.037 (1.539–2.697)	
Very Uncomfortable	1.616 (1.313–1.990)		1.812 (1.413–2.325)		2.088 (1.564–2.788)	
**Housing Issues**						
Very Comfortable	**Ref**	***p*-value**	**Ref**	***p*-value**	**Ref**	***p*-value**
Somewhat Comfortable	1.476 (1.214–1.794)	<0.001 *	1.377 (1.084–1.750)	<0.001 *	1.729 (1.294–2.310)	<0.001 *
Somewhat Uncomfortable	1.753 (1.429–2.151)		1.484 (1.152–1.911)		1.952 (1.443–2.638)	
Very Uncomfortable	1.996 (1.638–2.432)		1.942 (1.533–2.460)		2.672 (2.017–3.540)	

* *p*-value significant at <0.001 based on chi-squared test. ** aOR adjusted for participant demographic characteristics listed in Table 1. *** Reference category.

## Data Availability

Data were analyzed from the 2022 HINTS Survey 6 and are publicly available at https://hints.cancer.gov/.

## Public Involvement Statement

No public involvement in any aspect of this research.

## Guidelines and Standards Statement

This retrospective cross-sectional study was drafted against the STROBE guidelines.
